# Model test study on the effect of dynamic compaction under low water content

**DOI:** 10.1371/journal.pone.0253981

**Published:** 2021-06-29

**Authors:** Xiaoshuang Zhang, Min Wang, Yunshan Han

**Affiliations:** School of Science, North University of China, Taiyuan, China; Al Mansour University College-Baghdad-Iraq, IRAQ

## Abstract

Dynamic compaction is a cost-effective foundation treatment technology, that is widely used in various types and conditions of foundations. However, due to the limitation of natural conditions (water content between 3% and 8%) in north-western China, it is difficult to meet the requirements of the optimal water content during dynamic compaction. To better treat a foundation with a low water content, a series of model tests were carried out by using homemade test equipment to study the influence of the ramming energy and η value on the efficiency of dynamic compaction under a low water content. The results showed that the improvement of the energy level could compensate for the poor effect of dynamic compaction caused by a low water content in arid regions. Compared with that at the optimal water content, the efficiency of dynamic compaction was 58.1% to 66.2% at a low water content and excited the optimal energy level. Increasing the η value was also beneficial to improving the effect of dynamic compaction. Hence, the optimal energy level combined with the appropriate η value is of great merit in treating the foundation of arid regions by using the dynamic compaction method, which provides new parameter suggestions and engineering guidance for dynamic compaction construction in arid areas.

## 1 Introduction

The dynamic compaction method is to free fall the rammer (generally 100–400 kN) from a height (generally 6–40 m) to form shock waves and dynamic stress in the foundation soil so that the foundation soil can reach a high density and have good mechanical properties [1]. Due to the wide range of applications, simple equipment, high economic benefit and significant reinforcement effects, the dynamic compaction method has been widely used in practical engineering.

Researchers at home and abroad have mainly focused on the following aspects of dynamic compaction for a large amount of experimental research and theoretical analysis. The influence of water content on dynamic compaction efficiency at six field test cells was studied. It showed that the optimum water content and the maximum dry density were similar to the laboratory results [2]. The dynamic compaction effects in different soils were reported. The mechanics of dynamic compaction in dry sand were studied by using finite method and centrifuge model tests. The results showed that stress wave attenuation and improvement effects were realistically predicted [3]. The evaluation method of bearing capacity was studied to determine the coefficient and deformation modulus of composite foundation by dynamic tamping replacement method in the cold salt lake area [4]. The applicability and effect of dynamic compaction in collapsible loess area were also studied by field test [5]. The applicability and effectiveness of dynamic compaction were analysed in other foundation conditions by comparing the physical and mechanical indexes before and after dynamic compaction from various tests [6–9]. The effects of various dynamic compaction parameters on the mechanism of dynamic compaction were compared and analysed. To estimate the effective improvement range under dynamic compaction, the influences of tamping times, energy level, tamping distance, tamper radius and drop momentum on the relative degree of improvement were investigated [10]. It is found that the improvement depth and the improvement of the weak zone are highly correlated with drop energy and drop momentum, while the influence of the drop number and tamper radius is relatively smaller. The effects of blow numbers on the shear strength characteristics of loess were studied [11]. It was concluded that the shear strength of loess was basically controlled by its microstructural state. The lateral dynamic compaction on the slop was also studied by laboratory model tests [12]. Tamper energy and the distance from the point of impact to the edge of the slope have a great effect on increasing the bearing capacity of the strip foundation. The dynamic compaction method could combine with the chemical electroosmosis method, wellpoint precipitation method and other methods. It could effectively solved the problem of the poor effect of the dynamic compaction in strengthening individual soil foundations, providing new methods and ideas for the treatment of various types of foundation soil by the dynamic compaction method [13–15].

The above research work has laid the foundation for the extensive application of the dynamic compaction method in foundation treatment. However, in north-western China, collapsible loess is widely distributed, and water resources are scarce. The special climate makes the natural water content of the soil (3%-8%) generally lower than the required value of the optimal water content for dynamic compaction in the "Standard for Building Construction in Collapsible Loess Regions (GB50025-2018)" [16]. Moreover, in arid areas, the engineering cost of satisfying the optimal water content during dynamic compaction is very high. Hence, it is inevitable to study how to strengthen the effect of dynamic compaction of the foundation under low water content. Regrettably, almost no researchers have paid attention to the effect of dynamic compaction in arid areas.

In this paper, the optimal water content of soil in the test area was determined by a compaction test, and a series of dynamic compaction model tests were carried out by using homemade test equipment to study the influence of the energy level and η value (the ratio of the hammer weight to drop distance) on the efficiency of dynamic compaction under a low water content. This is of guiding significance to the economic construction of dynamic compaction in the reinforcement of low moisture foundations.

## 2 Laboratory model test research

### 2.1 Test soil samples

**Fig 1:**
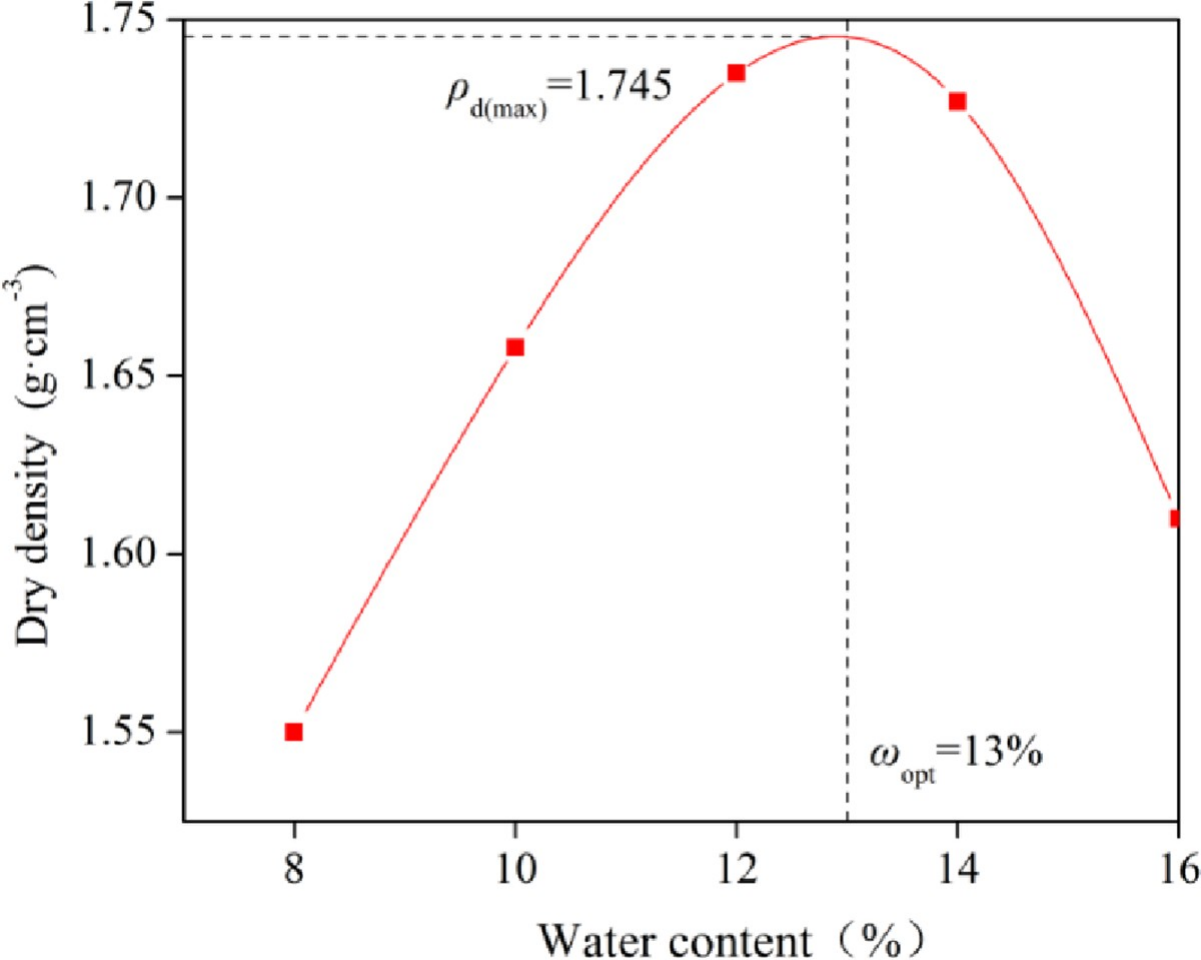
Compaction test curve.

The soil samples were taken from the North University of China. According to the Technical Specification of Dynamic Consolidation to Ground Treatment (CECS 279: 2010), it is advisable to humidify the soil close to the optimum water content when the natural water content of soil is less than 10% [17]. The optimum water content of the soil sample was obtained by a heavy compaction test (Fig 1). According to the provisions of the Standard for geotechnical testing method (GB/T 50123–2019) [18], five samples with different water content were prepared, and the difference between two adjacent water contents was 2%. The water content and dry density of each sample were calculated after compaction according to the requirements, and the compaction curve was drawn. The vertical axis corresponding to the peak point of the curve was the maximum dry density, and the corresponding abscissa was the optimal water content. The basic physical properties of the soil samples are shown in Table 1. By analysing the compaction test data, the maximum dry density *ρ*_*d*(max)_≈1.745 g/cm^3^ and the corresponding optimal water content *ω*_opt_≈13% of the soil sample were obtained.

**Table 1:** Physical parameters of the test soil.

Type	Initial water content ω (%)	Maximum dry density *ρ*_*d*(max)_(g/cm^3^)	Plastic limit ω_p_ (%)	Liquid limit ω_L_ (%)	Cohesion *c* (kPa)	Friction angle *φ*(°)	Specific gravity G_s_
Silty clay	6.94	1.745	13.2	26.8	9.5	20.4	2.70

### 2.2 Test device

**Fig 2:**
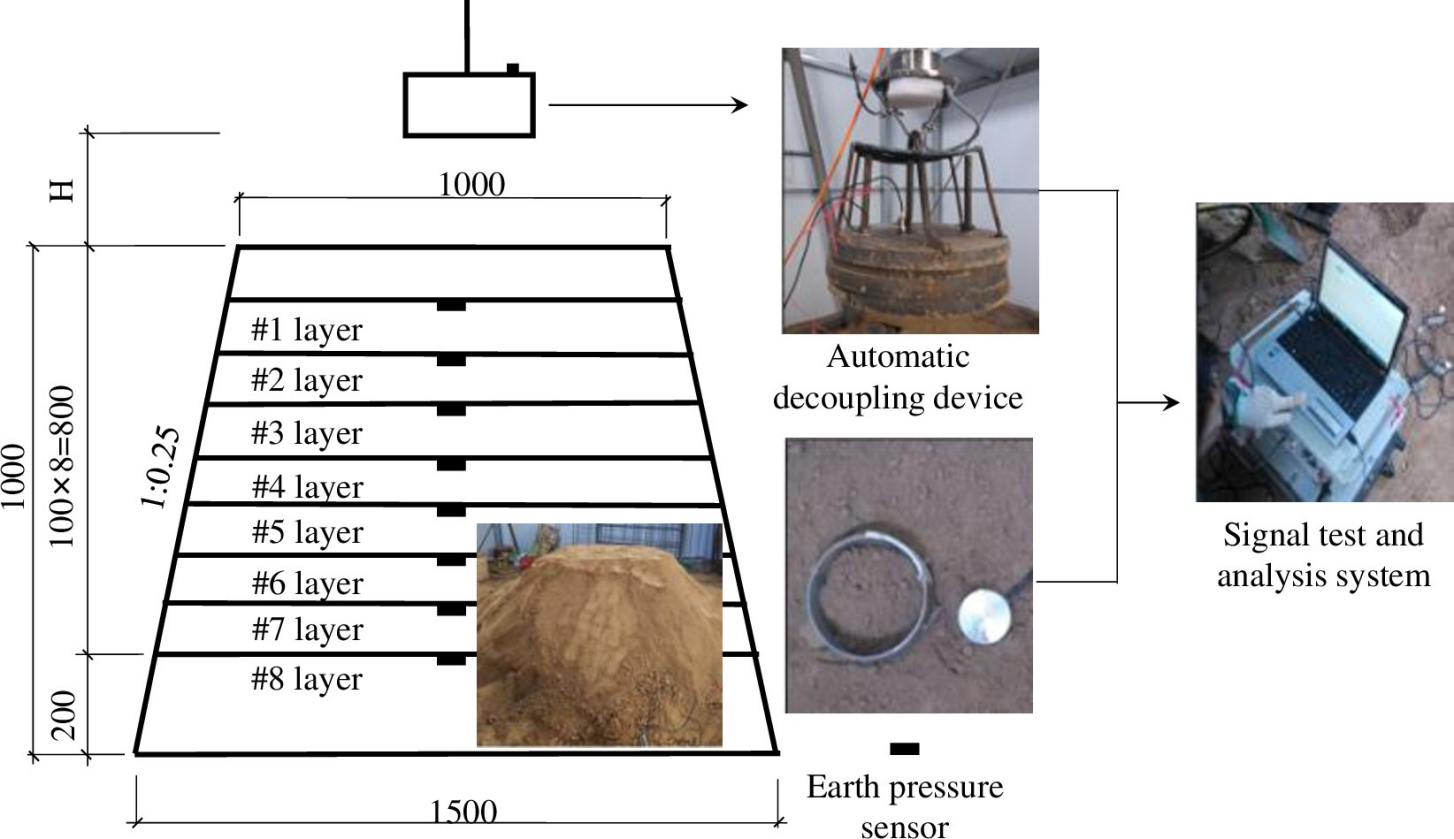
Schematic diagram of the model test.

To study the effect of dynamic compaction under a low water content, an indoor dynamic compaction model test device was designed according to the test requirements and conditions, as shown in Fig 2. The device consists of two parts: an automatic decoupling device and a data acquisition device.

#### 2.2.1 Automatic decoupling device of the rammer

The automatic decoupling device of the rammer uses an electromagnetic device to pull the rammer and uses the principle of power-off and demagnetization of silicon steel to make the rammer lose traction. Compared with the traditional decoupling device, the device improves the severe tilting situation after the rammer is dropped to the ground, which greatly improves the test accuracy.

**Fig 3:**
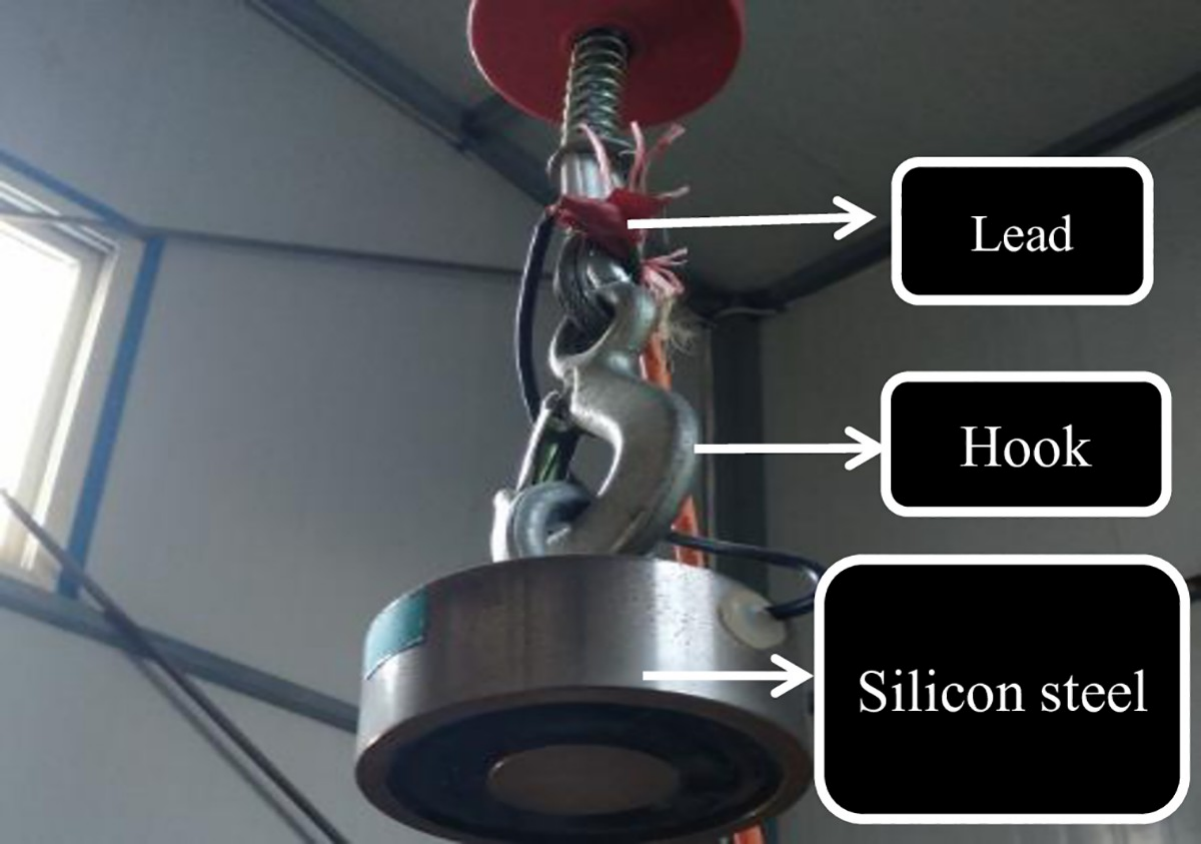
Electromagnetic self-weight decoupling device.

The automatic decoupling device is shown in Fig 3. The device is composed of a power supply, resistor, lead, hook and silicon steel.

#### 2.2.2 Data acquisition device

The settlement is measured directly by the tower ruler. The acceleration is measured by the piezoelectric acceleration sensor, and the dynamic stress is measured by the earth pressure sensor. The durability of the earth pressure sensor is measured and calibrated before use. The DHDAS(5956_NET) signal test and analysis system are adopted in the signal acquisition equipment to measure and record the dynamic stress and acceleration in real time.

### 2.3 Test process

The soil meeting the test requirements after humidification was piled up. The stacking depth was 1000 mm, and the slope rate was 1:0.25. Each layer was filled and compacted, and then, the earth pressure box was buried layer by layer as required until the slope was completed. The rammer was lifted to the required height of the scheme. After commissioning the data acquisition equipment, tamping was performed. After the tamping hammer was stable, the settlement was measured, recorded and saved.

### 2.4 Test conditions

#### 2.4.1 Determination of the similarity coefficient

Dimensional analysis of each parameter based on the similarity theory and π theorem obtained a weight similarity coefficient of *C_W_* = 512, a drop distance similarity coefficient of *C_h_* = 8, and a length ratio of $\lambda_{l}=\frac{l_{p}}{l_{m}}=8$ (*l*_p_ is the length of prototype, *l*_m_ is the length of model). The test material was the same as the prototype, taking a material similarity coefficient of *λ_ρ_* = 1. A comparison table of the prototype and test model of the dynamic compaction parameters was obtained, as shown in Table 2.

**Table 2:** Comparison of the prototype model parameters.

	Energy level E(kN·m)				Water content ω(%)	
Prototype	4000	6000	8000	10000	7	13
Model	0.977	1.465	1.953	2.441	7	13

#### 2.4.2 Test plan

To study the influence of energy level on the effect of dynamic compaction under a low water content, a series of tests were conducted, as shown in Table 3.

**Table 3:** Experimental scheme with different energy levels.

Group	Energy level E(kN·m)	Water content ω(%)	Hammer weight W(kg)	Fall distance H(m)
7%-4000	4000	7%	88.6	1.1
7%-6000	6000			1.65
7%-8000	8000			2.2
7%-10000	10000			2.76
13%-4000	4000	13%	88.6	1.1
13%-6000	6000			1.65
13%-8000	8000			2.2
13%-10000	10000			2.76

The energy level can be improved by increasing the hammer weight and drop distance. It is necessary to study the influence of the ratio of the hammer weight to drop distance on the effect of dynamic compaction under a low water content when the energy levels are the same. A series of tests were conducted, as shown in Table 4.

**Table 4:** Experimental scheme with different η values.

Group	Energy level E(kN·m)	Water content ω(%)	Hammer weight W(kg)	Fall distance H(m)	η value
7%-4000	4000	7%	57	1.71	33.53
7%-4000			88.6	1.1	80.55
7%-4000			122.6	0.8	153.25
7%-8000	8000	7%	57	3.42	16.76
7%-8000			88.6	2.2	40.27
7%-8000			122.6	1.6	76.63
13%-4000	4000	13%	57	1.71	33.53
13%-4000			88.6	1.1	80.55
13%-4000			122.6	0.8	153.25
13%-8000	8000	13%	57	3.42	16.76
13%-8000			88.6	2.2	40.27
13%-8000			122.6	1.6	76.63

## 3 Test results and analysis

### 3.1 Study on the effect of the energy level on dynamic compaction

#### 3.1.1 Analysis of settlement

**Fig 4:**
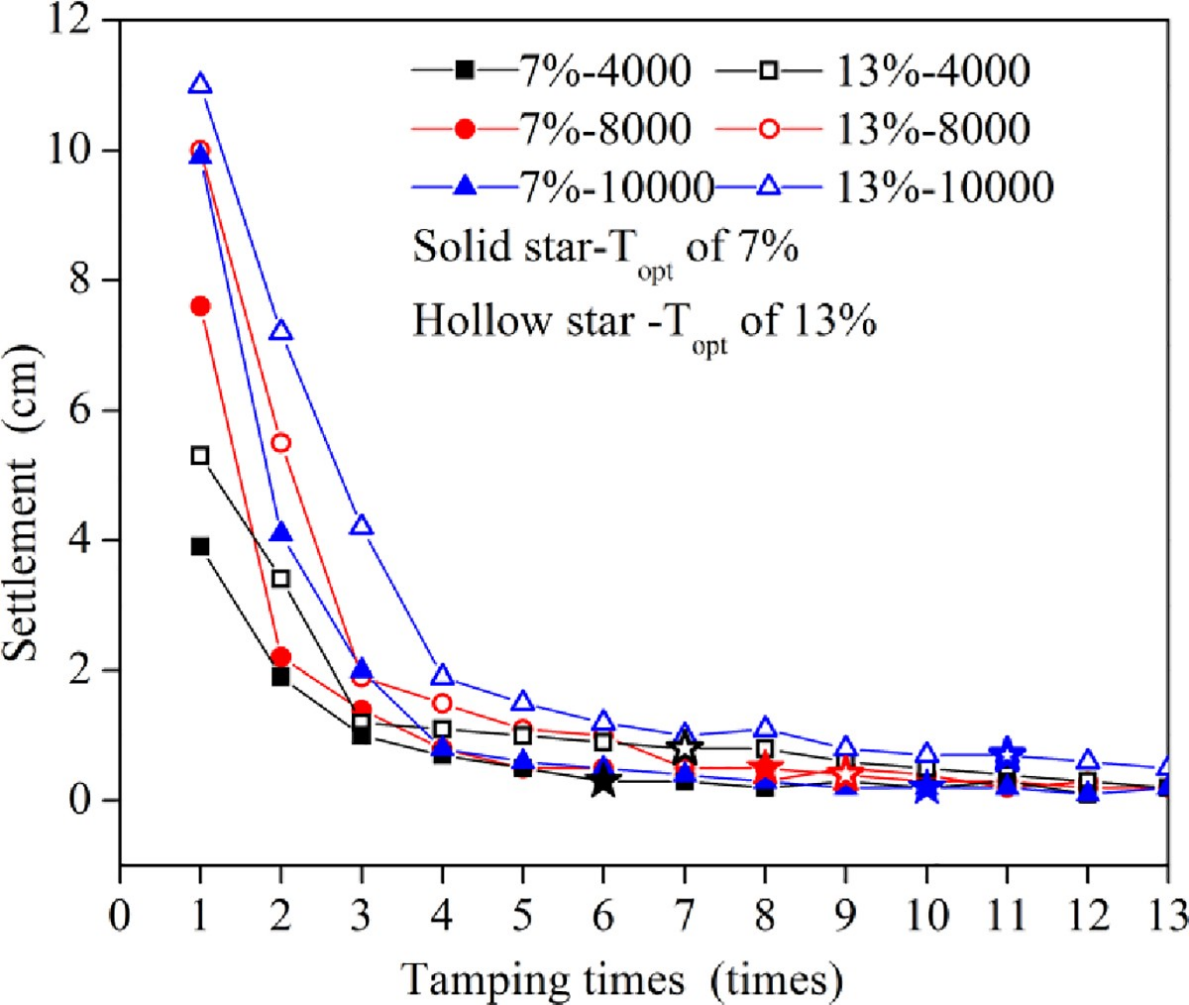
Curves of the settlement verses tamping times.

Fig 4 shows the curves of the settlement with the tamping times under different energy levels when the water content is the optimum water content of 13% and the low water content of 7%. As shown in Fig 4, the change trends of the settlements under the different conditions were the same, which decreased significantly with increasing tamping times and then gradually stabilized. In other words, there existed an optimal tamping time (T_opt_) (the starting point of stable segment where difference between two adjacent tamping amounts is less than 0.1cm). This deformation behavior agrees with the results reported in the references [11] and [19]. At the same energy level, T_opt_ decreases as the water content decreases. With the same water content, T_opt_ increased with increasing energy levels.

**Fig 5:**
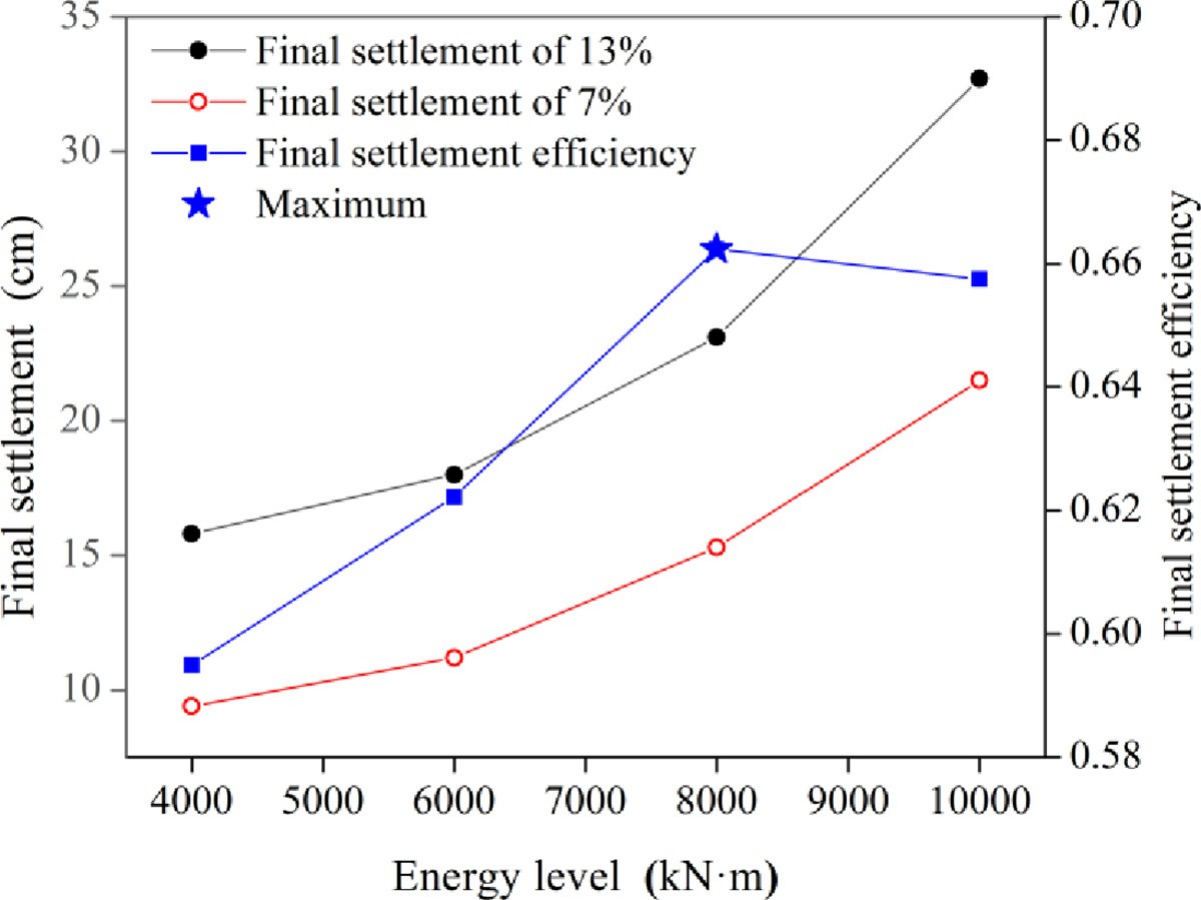
Curves of the final settlement and final settlement efficiency of dynamic compaction verses the energy level.

Fig 5 shows the trend of the final settlement and final settlement efficiency of dynamic compaction with the energy level under different water content conditions. The final settlement efficiency of dynamic compaction is the ratio of the final settlement under a low water content to that under an optimal water content. The final settlement increased significantly with increasing compaction energy, resulting in a better reinforcement effect. The final settlement efficiency at different energy levels varied from 58.1% to 66.2%, which is consistent with the conclusion reported by Rollins [2]. When the energy level was lower than 8000 kN·m, the final settlement efficiency improved as the energy level increased. However, when the energy level was higher than 8000 kN·m, the final settlement efficiency decreased, indicating that the energy level of 8000 kN·m was the optimum value at a low water contents. Hence, improving the energy level can effectively enhance the dynamic compaction effect, but an energy level that is too high easily causes waste.

**Fig 6:**
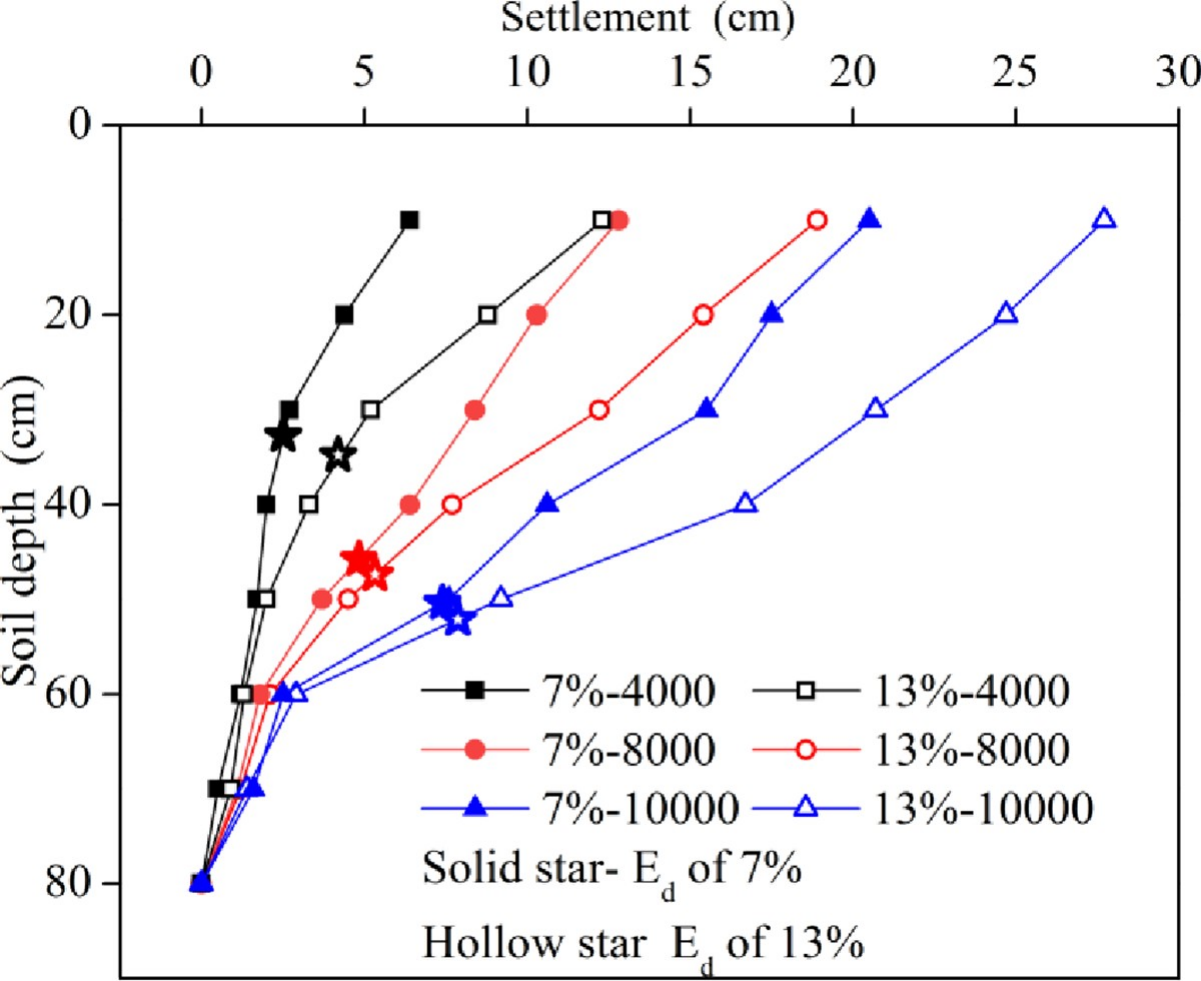
Curves of settlement verses the soil depth.

Fig 6 shows the curves of settlements with depth under different working conditions. Under various working conditions, the changing trends of layer settlements caused by the energy level or water content were the same; both decreased significantly with increasing soil depth and were close to zero at the bottom, indicating the existence of an effective reinforcement depth for dynamic compaction. When the water content was the same, the settlement at the same depth remarkably accelerated with the addition of the energy level. The effective reinforcement depth (E_d_: the depth of the foundation bearing capacity, deformation index, compactness, and other physical and mechanical indexes that meet the design requirements after the foundation soil is subjected to dynamic compaction) also increased as the energy level increased, indicating the improvement of the compaction effect. In addition, when the energy level was the same, the settlement at the same depth for the soil with a low water content of 7% was noteworthy less than that for the soil with the optimal water content of 13%. The variation in the effective reinforcement depth was the same as that of the settlement.

#### 3.1.2 Analysis of the peak acceleration

**Fig 7:**
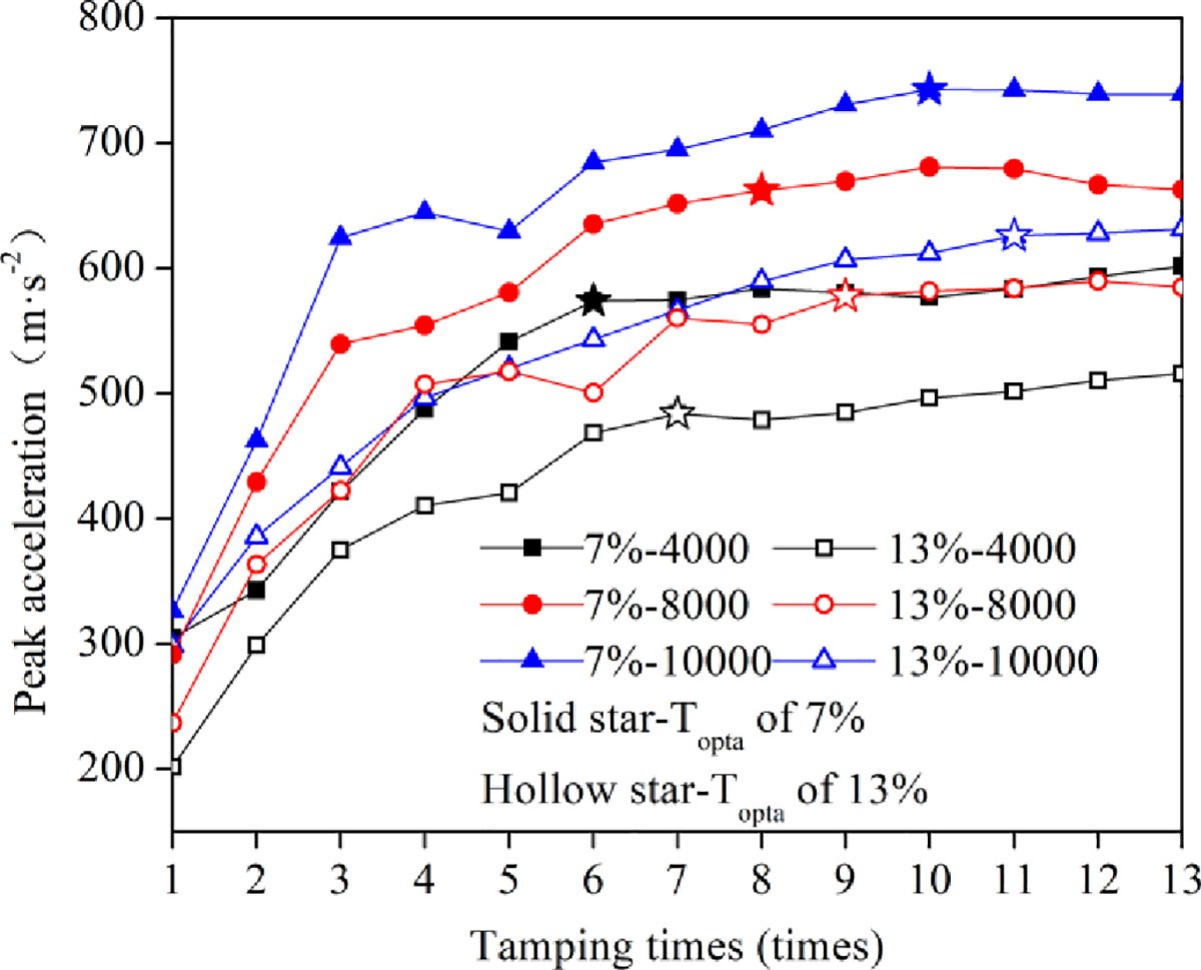
Curves of the peak acceleration verses tamping times.

Fig 7 shows the curves of the peak acceleration as a function of the tamping time. As shown in Fig 7, the peak acceleration increased significantly and then gradually with increasing tamping time. Moreover, the water content or energy level had the same effect on optimal tamping time T_opta_ (the starting point of stable segment where difference between two adjacent peak accelerations is less than 0.1cm). T_opta_ increased with increasing water content or energy level, which was basically the same as the change in settlement, indicating that the peak acceleration could be used as a parameter to reflect the effect of dynamic compaction. When the energy level was constant, the peak acceleration difference between the low water content and optimal water content first increased and then stabilized with increasing tamping times.

**Fig 8:**
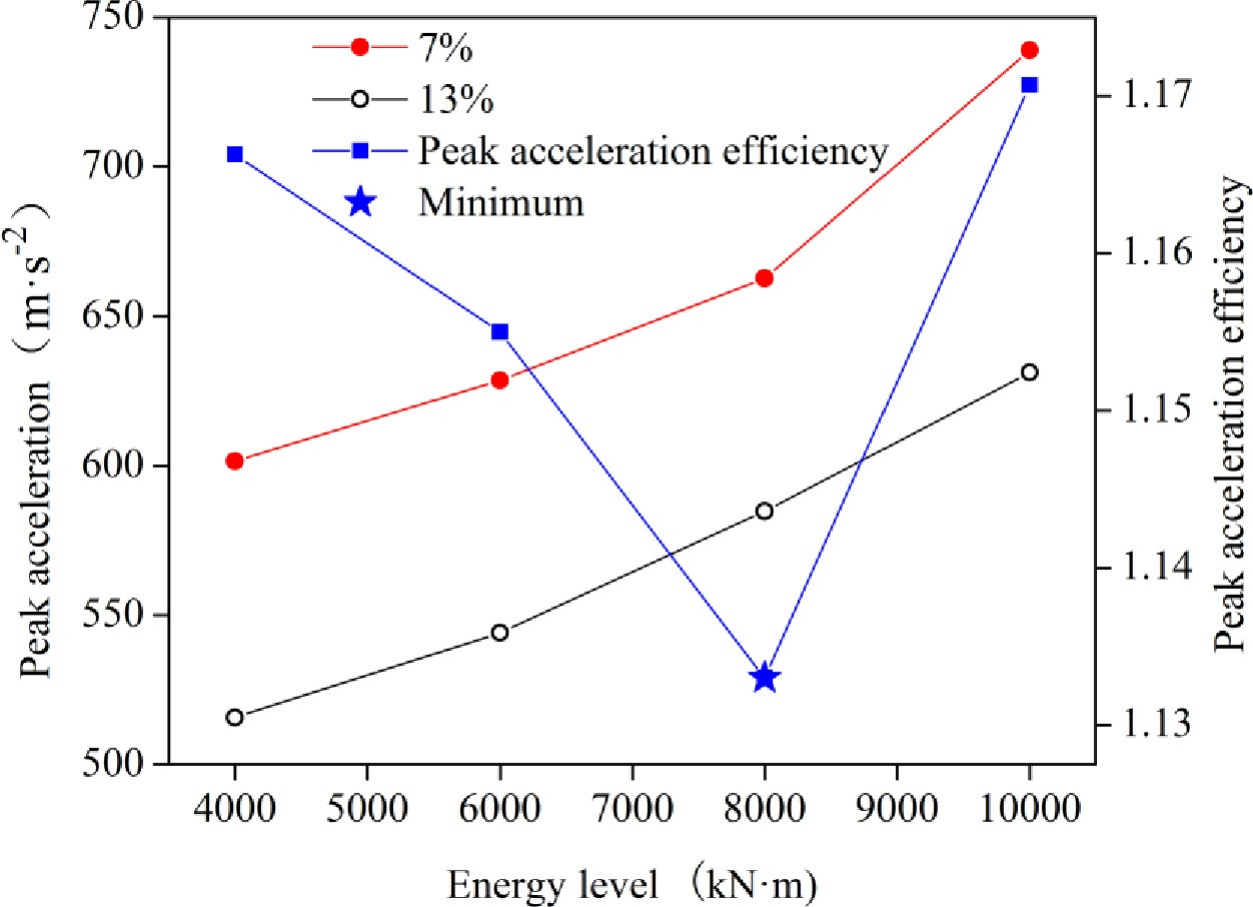
Curves of the final peak acceleration and E_a_ verses the energy level.

Fig 8 shows the final peak acceleration and final peak acceleration efficiency of dynamic compaction with the energy level under different water content conditions. The final peak acceleration efficiency of dynamic compaction is the ratio of the final peak acceleration under a low water content to that under an optimal water content. The final peak acceleration of dynamic compaction with a low water content was greater than that of the optimal water content at the same energy level. With the same water content, the final peak acceleration increased linearly with increasing energy level. In addition, when the energy level was lower than 8000 kN·m, the final peak acceleration efficiency decreased with the increase of the energy level; while, when the energy level exceeded 8000 kN·m, the final peak acceleration efficiency showed an opposite trend, which reflected the difference of the energy transformation when the soil reached the stable state during dynamic compaction. The energy conversion rate and the level of utilization are inversely proportional to the final peak acceleration efficiency. The smallest final peak acceleration efficiency located at 8000 kN·m was the optimal energy level, giving guiding significance for the actual construction of the project.

#### 3.1.3 Analysis of the dynamic stress

**Fig 9:**
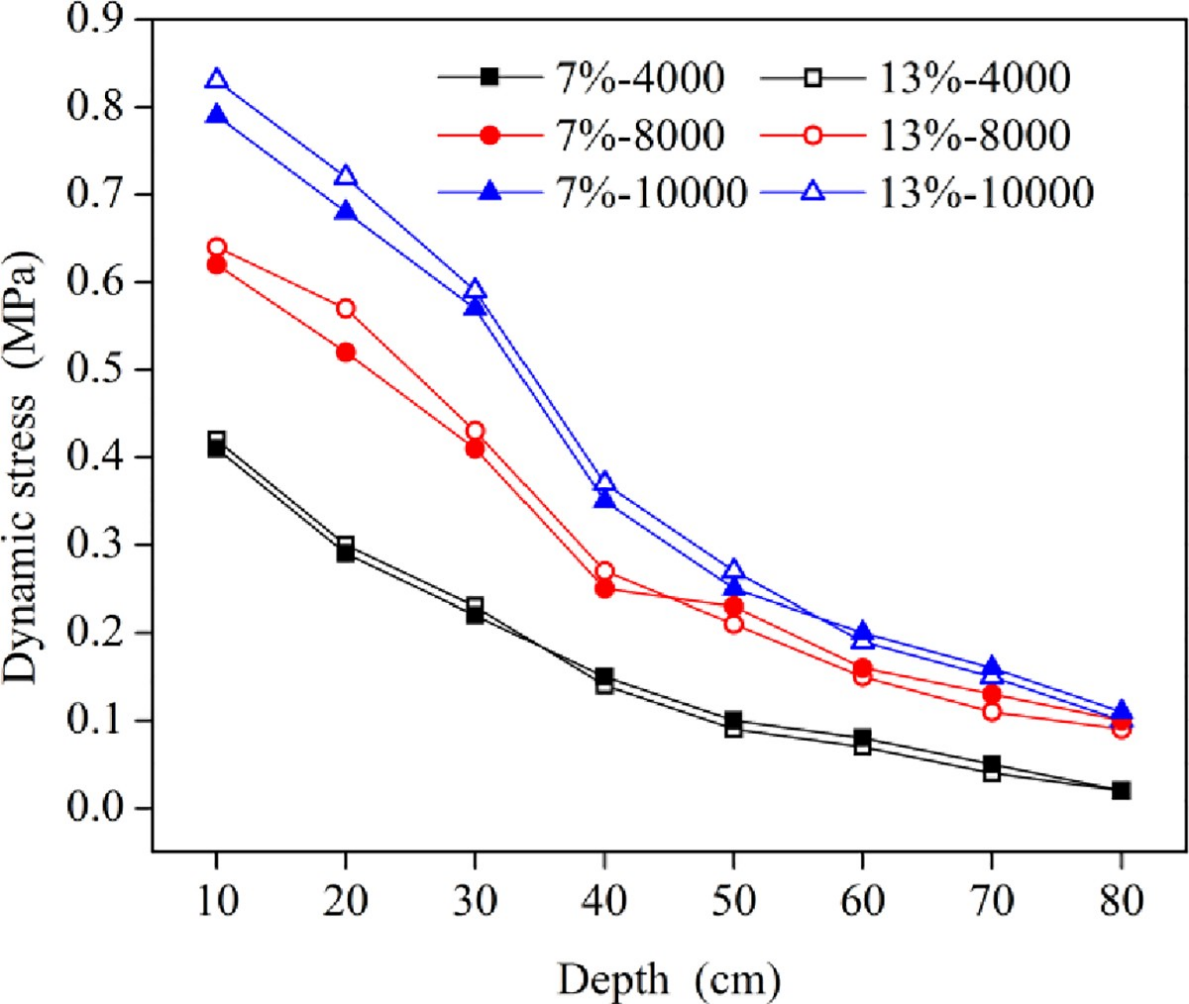
Variation curves of the dynamic stress verses different depths.

Fig 9 shows the curves of the dynamic stress with depth at the thirteenth tamping time. With increasing soil depth, the dynamic stress gradually decreased when the energy level increased. At the same energy level, the dynamic stress for the soil with the optimal water content and the low water content was almost small, indicating that the water content had little effect on the dynamic stress. Under the same water content, when the depth was less than 40 cm, the dynamic stress decreased significantly, and the rate of decrease accelerated with increasing energy level. When the depth was larger than 40 cm, the reduction in dynamic stress gradually slowed down, verifying that the effective reinforcement depth was located in this interval.

### 3.2 Study on the effect of η on dynamic compaction at a low water content

#### 3.2.1 Analysis of settlement

**Fig 10:**
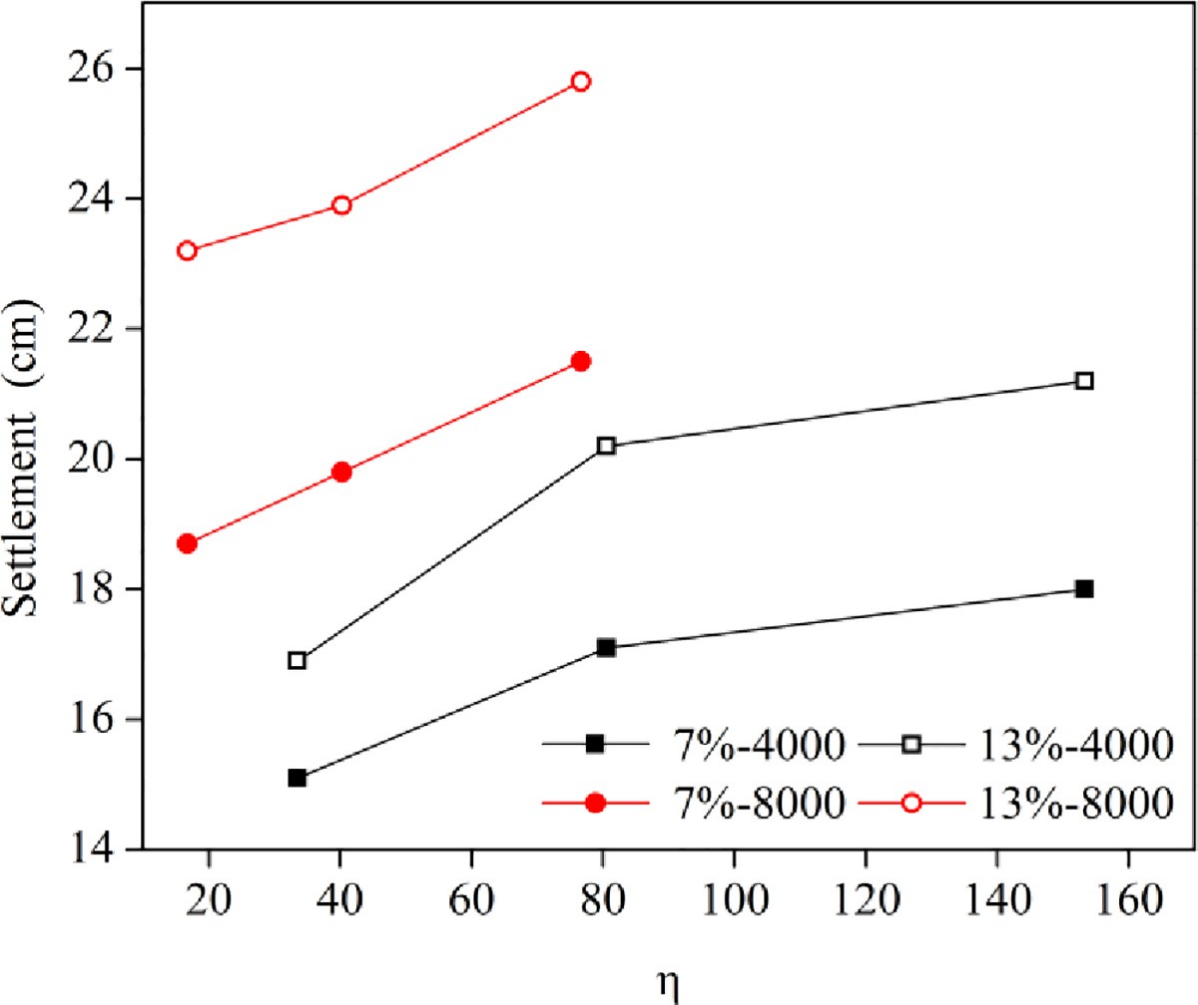
Curves of the final settlement verses the η value.

Fig 10 shows the variation of the final settlement with respect to the η value. The final settlement as a whole increased significantly with the η value, resulting in a better reinforcement effect. The similar trend has been reported [20,21]. The final settlement for the soil with a low water content of 7% was significantly less than that for the soil with the optimal water content of 13% under the same energy level, and the influence of the water content on the final settlement remained nearly the same with increasing η values. In addition, the influence of the energy level on final settlement was consistent with that of water content. However, the variation of the final settlement caused by the energy level was greater than that of the water content, indicating that the improvement of energy level could make up for the poor effect of dynamic compaction caused by a low water content in an arid region.

**Fig 11:**
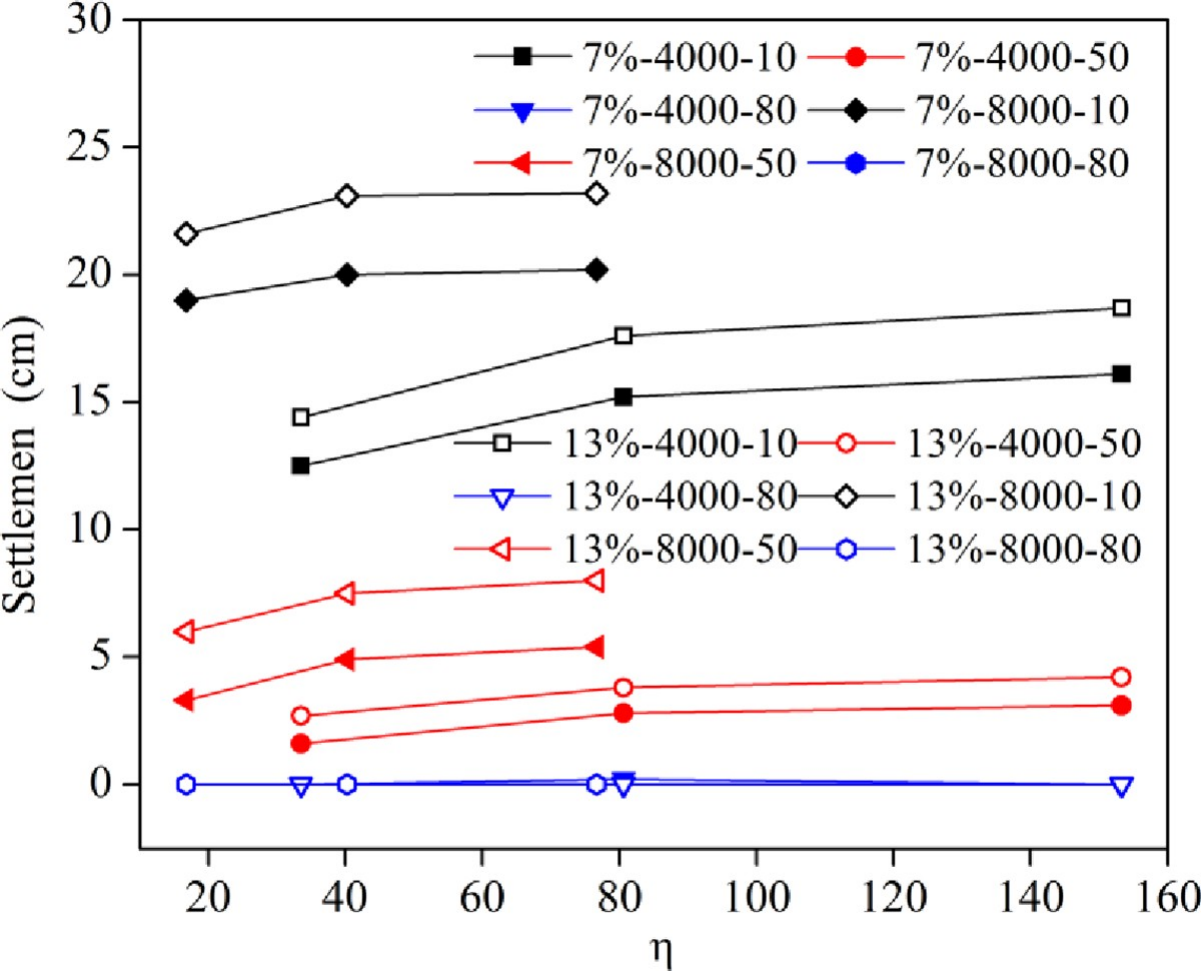
Change curves of the layer settlement verses the η value.

Fig 11 shows the curves of the sedimentation of some layers with respect to the η value. Under the same working conditions, the settlement of each layer increased slightly with increasing η values except for the settlement of nearly zero at the bottom. When the η value and the depth were the same, the effect of the energy level on the layer settlement was obviously greater than that of the water content.

#### 3.2.2 Analysis of the peak acceleration

**Fig 12:**
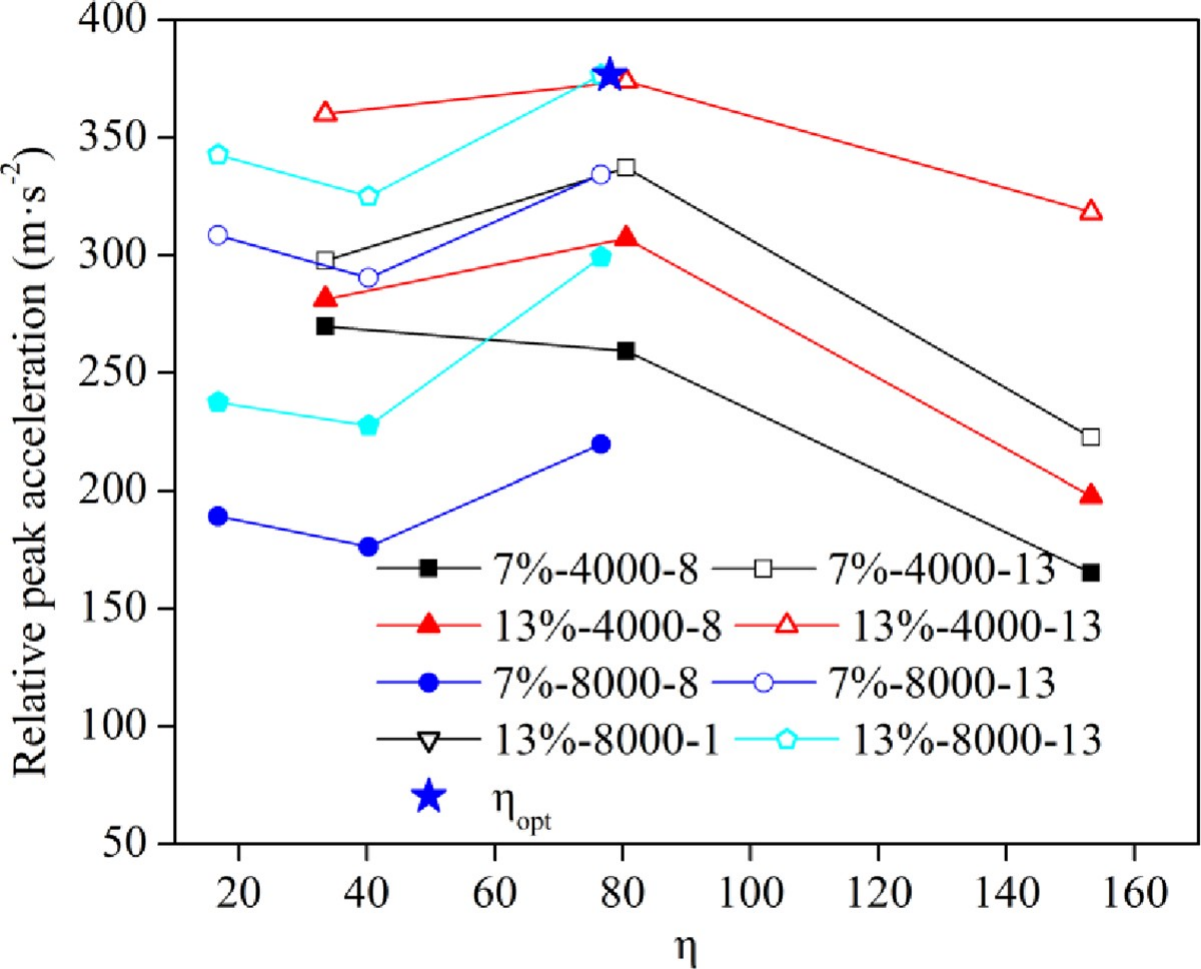
Curves of the relative peak acceleration for different tamping times.

Fig 12 shows the curves of the relative peak acceleration with the η value for different tamping times. As the η value increased, the relative peak acceleration (increment of peak acceleration relative to the first tamping) increased first and then decreased at a low energy level, which was instead at a high energy level. When the η value was approximately 80, the relative peak acceleration at the high energy level and the low energy level was basically the same, which meant that all of the energy generated by the increase in the energy level was effectively used, indicating that the dynamic compaction efficiency was relatively highest at this η value (η_opt_). Hence, a reasonable range of this parameter value needs to be provided after actual engineering trial compaction.

#### 3.2.3 Analysis of the dynamic stress

**Fig 13:**
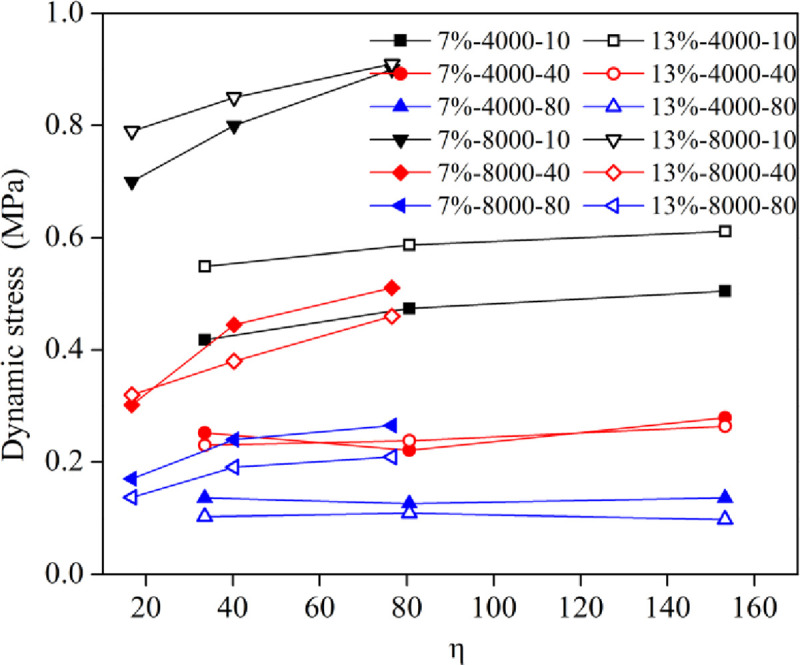
Variation curve of the dynamic stress at different depths.

Fig 13 shows the curves of the dynamic stress at the thirteenth tamping time with respect to the η value. The dynamic stress of each layer basically increased with increasing η values, but the dynamic stress increment of high energy level is significantly greater than that of low energy level. For a low energy level of 4000 kN•m, the difference in dynamic stress caused by the water content is large in the depth of 10 cm, but nearly same in the depths of 40 and 80 cm. However, for a low energy level of 8000 kN•m, the differences in dynamic stress caused by the water content in the depths of 10, 40 and 80 cm are both obvious and nearly remain the same with the increasing η values. This indicates that the increase of η value is beneficial to improve the dynamic compaction effect when the energy level is high.

## 4 Conclusions

Based on the tests and analyses undertaken, the following conclusions can be made:

1. The improvement of the energy level could compensate for the poor effect of dynamic compaction caused by a low water content in arid regions. Compared with the optimal water content, the efficiency of dynamic compaction was 58.1% to 66.2% at a low water content, which increased first and then decreased as the energy level increased, exciting the optimal energy level. Hence, it is not recommended to select ultrahigh energy when dynamic compaction is used to treat foundations in dry areas.
2. Increasing the η value was beneficial to improving the effect of dynamic compaction. When the η value is approximately 80 in this test, the dynamic compaction efficiency is relatively highest.
3. The optimal energy level combined with an appropriate η value is of great merit in dealing with the foundation of arid regions by using the dynamic compaction method, which provides new parameter suggestions and engineering guidance for dynamic compaction construction in arid areas.
